# Bacteria and What We Know about Them

**Published:** 1905-06

**Authors:** J. M. Fort

**Affiliations:** Paris, Texas


THE
### TEXAS MEDICAL JOURNAL.

ESTABLISHED JULY, 1885.

PUBLISHED MONTHLY.—SUBSCRIPTION $1.00 A YEAR.

VOL. XX. AUSTIN, JUNE, 1905. No. 12.

For Texas Medical Journal.

### Bacteria and What We Know About Them.

BY J. M. FORT, M. D., PARIS, TEXAS.

I shall begin this paper by asking and answering the question: "What are bacteria?"

Let me say, first, that bacteria are microscopic objects, infinitesimally small. They are beyond question a product of nature. And while there are hundreds of different species of them, there are only three general forms, namely, the spheres, the rods and the spirals. These different forms have been compared to billiard balls, lead pencils and corkscrews. These spheres may be large or small, and may group themselves in various ways. The rods may be long or short, thick or slender. The spirals may be loosely or tightly coiled and may have only one or two or may have many coils, and they may be flexible or rigid. In size they vary, many of them being the fraction of a millionth of an inch in diameter.

The most characteristic feature of the bacteria is their mode of growth or multiplication, i. e., by division or fission. In this method of growth they differ from yeasts, which increase in quantity by budding. And, while all bacteria multiply by segmentation or cleavage, they have different modes of doing so. While all bacteria multiply by division, certain differences in details produce rather striking differences in the results. Take the spherical forms. We find that some species divide, as described, into two and each of which in turn divides in opposite directions, called micrococcus. Other species divide in only one direction. Frequently they do not separate after dividing, but remain attached. Each, however, elongates and divides again, but all still remain attached. In this way they form long strings of spheres like beads. These are called streptococci. Other species divide first in one direction, then at right angles to the first division, and a third division follows at right angles to the plane of the first two, thus producing solid groups of fours, eights and sixteens. These are called sarcina.

The rod-shaped bacteria also differ somewhat, but to a less extent than the species just named. They divide generally at right angles to their longest diameter. Some of these rod-shaped bacilli separate immediately after division and put in an appearance as short, separate rods, while others remain attached after division and form long chains. At other times they elongate and form long threads —now and then they divide lengthwise, but this is seldom.

In the spiral forms we find they divide into short rods, or long, crooked chains or filaments, which seem to be fragile and easily break into short sections.

REPRODUCTION.

The rapidity of reproduction makes bacteria formidable. Some of the species have been watched under the microscope and found to divide every half hour. In one day each bacterium would produce sixteen million five hundred thousand. In two days two hundred and eighty million, which would form a pint of bacteria weighing not less than a pound. At the end of the third day they would reach forty-seven trillions, which would weigh something like a million pounds. Long before the offspring reach the millions, however, their multiplication is checked either by lack of food or by the accumulation of their own excreted products, which are injurious to them.

These figures show faintly what an unlimited power of multiplication these organisms have. They show us, too, that we are dealing with forces of almost unlimited extent.

While bacteria are simple in form, there are slight differences in them by which they may be distinguished; for instance, the rod bacillus is some times very blunt at the ends, as though they were cut square across. Some again are tapering, sometimes they are surrounded by a thin layer of some gelatinous material which forms what is called a capsule. This capsule may prevent their separation and form a chain. Sometimes this gelatinous fluid forms them into clusters.

These different appearances serve as one of the characteristics for distinguishing different species of bacteria.

In addition to their rapid reproduction by segmentation, many species of bacteria have another method, namely, by means of spores. These spores are oval bits of bacterial protoplasm capable of resisting adverse conditions which would destroy the ordinary bacteria.

They arise among bacteria in two different methods. The endogenous spores arise inside the rods of the spiral form; they break out of the rod and the rod producing them as a rule dies. The other method in some bacteria is as follows: The protoplasm in the large threads breaks into very minute spherical bodies, which find exit from the thread. Spore formation also serves as one of the modes of distinguishing the different species. These spores serve to keep the species alive under adverse circumstances. Some of them are capable of enduring a heat of 350 degrees F. without loss of vitality.

Many bacteria have flagella, or what in insects we call feelers; by these flagella they are enabled to swim. Some have one, some two and many a bunch of them.

It has been a question whether these bacteria are animal or vegetable. They have characteristics which ally them with both; for instance, their very common power of active, independent motion and their common habit of living upon complex bodies of foods, are animal characteristics, while on the other hand the absence of chlorofil coloring matter of plants, their general form, their method of growth, formation of threads and their method of spore-formation are like plants. The general belief, however, is that they form a group of plants by themselves, notwithstanding there is a great variety among them, so much so that bacteriologists have given them a generic name based upon their microscopic appearance, such as micrococcus, streptococcus, staphylococcus, sarcina, bacterium, bacillus and spirellum. These are all the names in common use applying to the ordinary bacteria. Of these bacteria a bacteriologist may find some sixty or seventy different varieties in a bit of cheese. To these common names a specific name is added, based upon some physiological character, as, for instance, "bacillus typhosus."

Where are these micro-organisms found?

There are no other plants or animals so universally found in nature. Their universality and great powers of multiplication render them of much importance in the domain of nature. They exist almost everywhere on the surface of the earth, and even below its surface to the depth of three or four feet. At the surface, especially if the soil is moist and full of organic matter. In such localities they are very abundant. Their number may range from a few hundred to a hundred million for every fifteen or twenty grains of soil. They are in all bodies of water at the surface and below the surface, even in the ocean. They are more abundant in running water than in stagnant water; all sediments of water are full of them. They are in the air and their number greater near the surface of the ground. And anything which tends to raise the dust increases the number of bacteria very greatly in the air. Every particle of decaying matter, no matter of what material it may be, is filled with them, for, in such places, they find their best food. The bodies of animals and man contain them in great abundance. They are in their mouths, stomachs and intestines. They are on the surface of their bodies, attached to their clothes, under their finger nails, on their hair and in every possible crevice and hiding place in the skin and in the outlets of all secretions. They do not appear in the healthy tissues of the body, in the blood, muscles, glands or any other organ. Secretions, such as urine and milk, are full of them. Not only higher animals, but all lower animals are covered with them. In short, wherever on the face of nature there is a lodging place for dust there will be found bacteria, and that in abundance.

There is a law governing these bacteria, and that is when they find food to their liking they, by increase in numbers, become a power. And, while this is true, it is no easy matter to starve them out. They and their spores can and do lie dormant, as when chilled with cold, for an incredible length of time and then be resuscitated and be as gay as ever.

These micro-organisms are now being extensively used in the arts and sciences in the making of linen, in jute and hemp, cocoanut fiber, sponges, leather preservations, maceration of skeletons, etc. In all dairy products and fermentative industries, such as vinegar, acetic, lactic, butyric acids, in the curing of tobacco and opium, etc.

Then we have another class of micro-organisms which play an important part in the growth and production of vegetable life. These are called nitrifying bacteria. They grow in the soil and feed upon soil ingredients. Wherever these bacteria live, grow and multiply they bring about important chemical changes. Their presence in many instances is conducive to our well-being.

Let me say further, in treating of bacteria as a whole, that nature appears limitless, for life processes have been going on in the world through countless centuries with a seemingly unimpaired vigor. And recent investigations prove beyond a doubt that at the very bottom we find this never-ending exhibition of vital power dependent upon certain activities of micro-organisms.

So thoroughly is this true that upon thorough investigation the continuance of life upon the surface of the earth would be impossible if bacterial action were checked for any considerable length of time. The life of the globe is, in short, dependent upon the activities of these micro-organisms.

While this in a sense may be true, at the same time they must be regarded as scavengers of no mean capacity. Every process of decay or putrefaction is set up by them. The whole process of decay of organic life is one in which bacteria play the most important part. All vegetable and woody matter, the process having been begun by moulds. This group of organisms, however, alone seem to be capable of attacking hard woody structure. The latter part of all decay in matter is carried on by bacteria alone. They are the sole agents which accomplish the decomposition of animal tissues. In a purely mechanical way, then, bacteria as decomposition agents are necessary to keep the surface of the earth fresh and unincumbered so that life can continue.

### DISEASE-PRODUCING GERMS.

Bacteria may be taken into the stomach by means of milk or water or other foods. These bacteria are not necessarily parasitic; that is, they do not feed upon the tissues of the body. On the contrary, they feed upon the milk or other articles of food before they are carried into the stomach. Now, so long as the individual is fed upon this bacteria-laden food, just so long will the poison excreted by the bacteria be absorbed into the system, and may produce a mild or severe disease, which may continue as long as this particular article of food is indulged in.

The most important disease of this class appears to be cholera infantum, so common among infants fed upon cow's milk in warm weather. We not infrequently meet with these poisoned cases from eating canned articles of food, also cheese or souse; in fact, we do not know at this time the extent of the troubles which are produced by bacteria of this sort. It is contended by some that these canned goods, when opened, come in contact with the air, which sets up a chemical action between the acid of the fruit and the tin vessel. Now, if this chemical action were purely a chemical action, it would have taken place before the can was opened. Then again, if this chemical action were the result of exposure to the air, there is nothing in the air to produce fermentation except the bacteria floating therein. If you admit that exposure to the air is necessary to the production of this chemical change, then you must admit it to be the bacilli which bring it about, and further that it is the absorption of the ptomaines which produces the disease.

However, in addition to these, there is a class of bacteria that is truly parasitic. This class not only invades the body, but lives and multiplies by feeding upon the tissues of the body instead of swallowed food.

However, it is difficult to draw a sharp line separating the two classes. The bacteria which cause diphtheria, for instance, do not really invade the body. They grow in the throat, attached to its walls, and are confined to this external location or to the superficial tissues. The bacillus is only found in the mouth and throat, and is practically confined to the so-called "false membrane." It never enters any of the tissues of the body, although attached to the mucous membrane. It grows vigorously in this membrane and there secretes and excretes extremely violent poisons, and it is the absorption of these poisons which produces the constitutional symptoms which we have in this disease. Very much the same is true of the bacillus which causes tetanus. This bacillus is commonly inoculated into the flesh of the victim by a wound made with some object which has been lying upon the earth where the bacillus lives. They grow readily after being inoculated, but they are localized at the point of the wound without invading the tissues to any extent. Among the poisons produced by this bacillus are several which have been separated and studied. Among them are some of the most violent poisons known.

It is but a step from these bacilli to the true parasites—the typhoid bacillus for instance. Typhoid fever is a disease produced by a parasitic bacillus which grows, or rather multiplies, in the glands of the intestines, and also in the tissues more extensively than the cholera germs. They do not invade the body generally, however, but become somewhat localized in special glands, namely, in the glands of the intestines, in the liver and spleen. They do not find very favorable conditions in the liver and spleen for they do not grow extensively in these glands. When found in the liver or spleen, they are found in small groups or centers, but not generally distributed through them. But, wherever they grow, they produce poison which has been called "typho-toxine," and it is this poison chiefly which gives rise to fever.

Quite a considerable number of the pathogenic bacilli are like the typhoid germ, more or less confined in their field of operation to special localities, i. e., instead of distributing themselves through the body, after they find entrance, they are restricted to certain organs.

The most common example of a parasite of this sort is the tuberculosis bacillus, the cause of consumption, scrofula, white swelling, lupus, etc. Although this bacillus is very common and is able to attack almost any organ in the body, it is usually very restricted in its growth. It may become localized in a small gland, a single joint, a small spot in the lung, or one or more of the mesenteric glands, or a single bone. Not infrequently the trouble is thus confined to such a small locality that nothing serious results. But, in other instances, the bacilli may after a time, slowly or maybe rapidly, distribute themselves from these centers, attacking more and more of the organs until perhaps fatal results follow in the end. Hence this disease is of slow progress.

We have another parasitic bacillus which is not thus confined, but which, as soon as it enters the system, produces a general infection, attacking the blood and perhaps nearly all the tissues simultaneously. Perhaps the most typical example of this sort or species of bacillus is the anthrax or malignant pustule, a disease fortunately rare in man. Here the bacilli multiply in the blood and very soon a general and fatal infection of the whole body arises, resulting from an abundance of the bacilli everywhere.

Some of the obscure diseases known as blood poisoning appear to be of the same general nature—these embrace septicemia, pyemia, gangrene, inflammation of wounds, abscesses, ulcers, erysipelas, etc.

In general, then, all the so-called germ diseases result from the action upon the body of poisons usuallly called ptomaines, produced by bacterial growth.

"Differences in the nature of these poisons produce differences in the character of the disease, and differences in the parasitic powers of the different species of bacteria produce wide differences in the course of the diseases and their relation to external phenomena."

While this subject is one of particular interest to the physician, it is perhaps of equal interest for him to understand what are nature's forces which are arrayed against these invaders. The human system makes a brave fight against these bacilli, especially the pathogenic species, after they have entered the human organism. The great host of species which are found in water, milk, air, in our mouths or clinging to our clothes or person, and which are almost omnipresent in nature, are capable of growing well enough in ordinary lifeless organic foods; but, just as soon as they succeed in finding entrance into livng human tissues, their growth is immediately checked by certain antiseptic agents which are poured upon them. Such bacteria are not, therefore, pathogenic germs or sources of trouble to human health.

A question: How do these pathogenic germs overcome the poisons which kill the ordinary bacteria, such as I have described above?

It is an undoubted fact that the first step in repelling these bacteria is to flood them with certain poisons which check their growth. In the blood and lymph of man and other animals there are present certain products which have a direct deleterious influence upon the growth of micro-organisms. Human flesh or human blood will furnish excellent food for them if the individual be dead, but living human flesh and blood in some way exert a repressing influence upon them which is fatal to the growth of a vast majority of species.

However, some few species are not thus destroyed bv the hostile agencies of the tissues of the animal, but are capable of multiplying or growing in the living body.

What are the forces arrayed against these invaders? The essential nature of the battle appears to be a production of poisons and counter-poisons.

The first step is to check them with certain poisons. Experiments made time and again have demonstrated the existence of these poisons in the blood of animals. Of their nature we know but little, but of their repressing influence upon bacterial growth we are sure. These poisons are called "alexines." They are products in the living tissues, although as to their method of production we are ignorant. By the aid of these poisons the body is able to prevent the growth of the vast majority of bacteria which get into its tissues.

Ordinarily, bacteria or micro-organisms are killed at once by this alexine product. When the pathogenic bacteria get into the body, they give rise to a product or a group of bodies called "lysines." These lysines are as mysterious to microscopists as the alexines. The lysines overcome the resistance which the body offers to the growth of bacteria, which gives the pathogenic germs an opportunity to get a foothold in the system, which soon produces the abnormal symptoms we call disease.

But now another of nature's reserved forces comes into play. This second method is inaugurated by a series of active cells in the blood, known as white blood corpuscles. They are minute bits of protoplasm present in the blood and lymph in large quantities. They are active, living cells, capable of locomotion and able to crawl out of the blood-vessels. They can and do take into their bodies small objects with which they come into contact. They act as scavengers. Very commonly they collect in great numbers in the region of the body where invading bacteria are found. These invading bacteria seem to exert a strong attraction upon them and at times they form a solid phalanx, completely surrounding the invading germs. Their collection at these points may make itself seen externally by the phenomenon we call inflammation.

There is no question but that these corpuscles engage in conflict with the bacteria when they thus surround them. It has been contended by some bacteriologists that these corpuscles actually take the bacteria into their bodies, swallow them as it were, and subsequently digest them. This idea gave rise to the theory of phagocytosis, and hence the corpuscles are called phagocytes. This idea, however, has been partially abandoned and it is now thought that the corpuscles secrete from their own bodies certain injurious products which act upon the bacteria much as do the alexines already mentioned. These two products, acting in conjunction, may entirely destroy the invading bacteria. After the bacteria are destroyed, the phagocytes load themselves with the dead bacteria and carry them to various parts of the body for elimination.

Not infrequently the corpuscles die in the struggle for mastery, and then may accumulate in the form of pus and seek an external outlet through the skin. This battle between the phagocytes and the bacteria goes on vigorously, but the victim may be entirely ignorant of it. He may not even know that he has been exposed to an attack.

In other cases the bacteria are too strong for the phagocytes and alexines. In that case they multiply rapidly and drive the phagocytes from the field of action. Under these circumstances the invading hosts can multiply unimpeded, and distribute themselves rapidly over the body and the disease rapidly follows. As the bacteria become more numerous, their poisonous products increase and begin to produce direct poisonous effects in the body. When this is the case, we may say that the stage of incubation has passed, and the disease now comes on and runs its course.

As a rule, the disease becomes more and more severe until a crisis is reached. Then, unless the poisoning is so severe that death takes place, the effects pass away and recovery takes place.

It may be asked: Why should not a germ disease always prove fatal?

If the bacteria thus take possession of the body and can and do grow there in spite of the warfare carried on between them and the alexines and phagocytes, why do they not continue to multiply until they produce sufficient poison to destroy the life of the individual? Such fatal results do, of course, occur, but by far the larger proportion of the cases recover.

As shown, for a time the multiplying bacteria have an unimpeded course, and often grow rapidly; but finally their increase is checked, their vigor impaired, their number diminished, and finally expelled fıom the body entirely.

Now here is introduced a new element in their growth, of which but little is known. It appears beyond a question that in the case of certain of these germ diseases the cells of the body, after a time, produce substances which serve as antidotes to the poisons by the bacteria during their growth in the body. In other words, they produce antitoxines.

That there is a cellular resistance to the action of the poisons of this class of parasitic bacteria, engendered during the progress of this class of diseases, there can be no doubt, as the fact is evidenced by the immunity afforded by one attack from a subsequent attack of the same disease.

This acquired immunity is not thoroughly understood by bacteriologists, but every day observation demonstrates its existence; it is a fact we can not gainsay.

Let me say, in conclusion: The majority of germ diseases are divided into three stages: First, the stage of incubation; second, the development of the disease; and, third, recovery; and, in the great majority of cases, immunity from second attack. And immunity from a subsequent attack is proof, beyond dispute, that the presence of these bacteria or parasites and their poisons has brought about a power of resistance in the cells of the body which did not exist there before. And this constitutes the safety of the patient in all this class of diseases.

---

There was a meeting of County and City Health Officers held at Austin May 22d upon call of State Health Officer Tabor. About sixty were present. Quarantine was the principal topic, except a paper by Dr. J. S. Lankford, of San Antonio, upon the necessity of teaching preventive medicine in the common schools, which met with much favor.

